# Behavioral and Cognitive Assessment in a Cohort of Term Small-for-Gestational-Age Children

**DOI:** 10.3390/children13010120

**Published:** 2026-01-13

**Authors:** Rossella Vitale, Annachiara Libraro, Francesca Cocciolo, Mariangela Chiarito, Emilia Matera, Maria Felicia Faienza

**Affiliations:** 1 Department of Precision and Regenerative Medicine and Ionian Area, University of Bari “Aldo Moro”, 70124 Bari, Italy; r.vitale7@studenti.uniba.it (R.V.); francescacocciolo15@gmail.com (F.C.); mariangela.chiarito@policlinico.ba.it (M.C.); emilia.matera@uniba.it (E.M.); 2 Department of Translational Biomedicine and Neuroscience, University of Bari “Aldo Moro”, 70124 Bari, Italy; annachiara.libraro@asl.brindisi.it

**Keywords:** small for gestational age, cognitive impairment, WISC-IV, intelligence quotient

## Abstract

**Highlights:**

**What is the main findings?**

• Term-born SGA children show a slightly reduced overall IQ with specific weaknesses in visuospatial abilities, attention, and processing speed, while verbal and memory skills remain relatively preserved.

**What is the implication of the main finding?**

• Early identification of deficits in perceptual reasoning and processing speed may guide timely cognitive and educational interventions, potentially improving long-term academic and social outcomes.

**Abstract:**

Background/Objectives: Children born small for gestational age (SGA) are at increased risk for impaired growth, metabolic disturbances, and neurodevelopmental difficulties. Although previous research has examined cognitive and behavioral outcomes in this population, findings remain inconsistent. Moreover, limited evidence is available regarding the potential effects of recombinant human growth hormone (rhGH) therapy on cognitive development. We aimed to assess cognitive performance, emotional–behavioral functioning, and neonatal predictors of neurocognitive outcomes in term SGA children compared with age- and sex-matched peers born appropriate for gestational age (AGA). We also explored potential differences in cognitive outcomes between rhGH-treated and untreated SGA children. Methods: A total of 18 term SGA children and 23 AGA controls underwent anthropometric measurements, biochemical evaluation, cognitive testing using the Wechsler Intelligence Scale for Children—Fourth Edition (WISC-IV), and behavioral assessment through the Child Behavior Checklist (CBCL). Birth weight, length, and head circumference were analyzed as potential predictors of cognitive performance. Results: SGA children demonstrated significantly lower Intelligence Quotient (IQ) scores than AGA peers, with marked weaknesses in Perceptual Reasoning index (PRI) and Processing Speed index (PSI), while Verbal Comprehension and Working Memory were preserved. They also exhibited higher internalizing behavioral symptoms, whereas externalizing behaviors did not differ between groups. Birth head circumference emerged as a strong predictor of PRI and a modest predictor of PSI. No associations were found between rhGH treatment parameters and cognitive outcomes. Larger longitudinal studies are needed to clarify how early growth restriction affects brain development and cognition and whether GH therapy influences these processes.

## 1. Introduction

Children born small for gestational age (SGA), defined as having a birth weight and/or length below the 3rd percentile (or −2 SDS), represent approximately 5–10% of all births. They constitute a population that requires careful follow-up, as they are at increased risk of experiencing several adverse outcomes, including impaired postnatal growth, cardio-metabolic complications, and altered neurodevelopment [1,2,3,4]. Most children born SGA demonstrate catch-up growth into the normal range between 6 months and 2–4 years of age. However, approximately 10–15% of SGA individuals fail to reach their full growth potential and remain short throughout childhood. In the majority of cases, this condition is not attributable to growth hormone (GH) deficiency, but rather to varying degrees of resistance within the GH–insulin-like growth factor 1 (IGF-1) signaling axis [5]. For these patients, recombinant human growth hormone (rhGH) therapy is indicated to promote linear growth. Cognitive impairment is another potential consequence of being born SGA, supported by neuroimaging evidence demonstrating widespread reductions in brain volume and cortical and subcortical surface area across multiple regions [6]. Some studies have shown that children born SGA at term are at increased risk of mild cognitive dysfunction and lower academic performance throughout childhood and adolescence [7,8]. However, other investigations have reported contrasting findings, noting no significant differences in total Intelligence Quotient (IQ) between SGA children and their Appropriate-for-Gestational-Age (AGA) peers [9,10]. A meta-analysis by Sacchi et al. reported that SGA children have significantly lower IQ scores than their AGA peers, with a mean difference of approximately −4.6 points [11]. In contrast, a systematic review by Taine et al. [12] highlighted that early postnatal catch-up growth—particularly within the first 6 months of life—is positively associated with favorable cognitive outcomes, including IQ, among children born SGA at term. Data on attention problems are more consistent, with several studies reporting a higher incidence of attention deficits among children born SGA than AGA peers [7,13,14]. Moreover, investigations focusing specifically on the language domain have documented a higher prevalence of language difficulties in this population [15,16], which may be explained by their lower performance in phonological coding and decoding tasks—skills that are essential for the development of reading and writing abilities [17]. Furthermore, behavioral difficulties, including a greater tendency toward anxiety and depressive symptoms have also been reported [18]. However, results derived from standardized cognitive tests and neuropsychiatric assessments in populations of children born SGA are highly heterogeneous due to differences in country of origin, cognitive and behavioral domains assessed, definitions of SGA, and age of the children at the time of the recruitment. In addition, although GH is known to play a key role in central nervous system development, at present, there is no evidence regarding the effects of rhGH treatment on cognitive development in SGA children.

Based on these considerations, the primary objective of this study was to evaluate neurocognitive performance as well as emotional and behavioral difficulties in a cohort of children born at term, comparing them with an age- and sex-matched cohort of AGA children. In addition, we assessed potential differences in cognitive performance between rhGH-treated SGA children and those untreated.

## 2. Materials and Methods

### 2.1. Subjects

This cross-sectional study included a cohort of 18 children born SGA at term, who were followed at the Endocrinology and Rare Endocrine Diseases Clinic of the University of Bari, and a control group of 23 children born AGA at term attending the Neuropsychiatric Clinic of the University of Bari for learning difficulties. In the control group the diagnosis of a neurodevelopmental disorder was ruled out through standardized clinical assessment. The exclusion criteria for both patients and controls included chromosomal or genetic syndromes, cognitive delay secondary to intrauterine or postnatal complications (such as asphyxia or brain injury), preterm birth, and neuropsychiatric disorders (e.g., Autism Spectrum Disorder and Attention-Deficit/Hyperactivity Disorder). Participants were consecutively recruited during routine follow-up visits.

The study protocol was approved by the Local Ethics Committee and parents or legal guardians of all participants provided written informed consent before inclusion. All procedures were carried out in accordance with the ethical standards of the institutional research committee and with the principles of the Declaration of Helsinki on Human Experimentation.

### 2.2. Clinical Data

Neonatal anthropometric data—including birth weight, length, and head circumference—were obtained from medical records for SGA subjects, and from anamnestic history for controls. All measurements were expressed as standard deviation scores (SDS), adjusted for gestational age and sex according to the Italian Neonatal Study (INeS) reference charts [19]. Children were classified as SGA if their birth weight and/or length were below −2 SDS or the 3rd percentile. AGA children were defined as those with birth weight and/or length greater than −2 SDS and up to +2 SDS.

At recruitment, anthropometric parameters (heigh, weight, Body mass index-BMI) were assessed for every patient and compared with the standard growth charts for the Italian population for age and sex [20] and expressed as SDS calculated using Growth Calculator^®^ Software version 3.0. Pubertal stages were assessed using the Tanner method [21]. Among rhGH treated SGA children, information regarding the age at start of therapy, and dosage was collected.

### 2.3. Biochemical Assessment

After an overnight fast, peripheral venous blood samples were collected from patients and controls into serum-separating tubes. Serum concentrations of total cholesterol (TC, mg/dL), low-density lipoprotein cholesterol (LDL), high-density lipoprotein cholesterol (HDL), and triglycerides (TG) were measured as part of a complete lipid panel using a spectrophotometric method. These assessments are included in the routine follow-up of children born SGA. Thyroid function was evaluated by measuring thyroid-stimulating hormone (TSH) and free thyroxine (FT4) using a chemiluminescence method to exclude other potential causes of neurocognitive impairment and reduced linear growth.

### 2.4. Cognitive Performance

Patients underwent cognitive assessment using the Wechsler Intelligence Scale for Children–Fourth Edition (WISC-IV), administered to children aged 6 years to 16 years and 11 months [22]. The WISC-IV is a widely used instrument for formal cognitive evaluation and provides a total IQ as well as four cognitive domain indices: the Verbal Comprehension Index (VCI), Perceptual Reasoning Index (PRI), Working Memory Index (WMI), and Processing Speed Index (PSI).

The VCI evaluates the child’s ability to formulate and use verbal concepts, including listening to instructions, retrieving learned information, reasoning, and verbally expressing responses. The PRI measures nonverbal reasoning and fluid intelligence—abilities minimally influenced by cultural or educational background—and assesses the capacity to analyze problems, apply visuomotor and visuospatial skills, plan, and generate and evaluate solutions. The WMI assesses the ability to encode new information, store it in short-term memory, sustain attention, and mentally manipulate information to derive solutions. The PSI evaluates the child’s capacity to maintain focused attention, rapidly scan visual stimuli, and process information efficiently [22]. Composite scores can also be calculated as an option. Evidence indicates that the WISC-IV demonstrates adequate internal consistency and construct validity for estimating general intellectual ability [23].

### 2.5. Psycological Evaluation

Psychological evaluation was conducted through clinical observation performed by trained psychologists and child neuropsychiatrists during in-person assessment sessions, together with the administration of standardized parent-report questionnaires, including the Child Behavior Checklist (CBCL), in both study groups [24]. The CBCL for children aged 6 to 18 years is a parent-reported questionnaire consisting of 113 items designed to assess emotional and behavioral difficulties. The instrument demonstrates good psychometric properties, with an average sensitivity of 0.63 and an average specificity of 0.84. Scoring was performed for the composite clinical scales—Internalizing and Externalizing. Raw scores were converted into T-scores, with T-scores ≥ 65 indicating clinically significant elevations and T-scores of 60–64 reflecting borderline or subclinical concerns. The Internalizing scale evaluates symptoms such as anxiety, depression, and social withdrawal, whereas the Externalizing scale assesses disruptive behaviors, aggression, and defiance. In addition, a psychological anamnesis was collected from caregivers at the time of CBCL administration to gather parental perspectives on the children’s difficulties.

### 2.6. Statistical Analysis

Statistical analyses were performed using SigmaPlot^®^ Software, version 12 for Windows. Descriptive statistics were computed for all demographic, anthropometric, and cognitive variables. The distribution of continuous variables was assessed using the Shapiro–Wilk test; since most variables were not normally distributed, non-parametric tests were applied when appropriate. Differences between groups (AGA, SGA-treated, SGA-untreated) were evaluated using the Kruskal–Wallis one-way analysis of variance on ranks, followed by pairwise post hoc comparisons with Bonferroni correction. Independent-sample comparisons within SGA subgroups were conducted using Student’s *t*-test or Mann–Whitney U test as appropriate.

Associations between continuous variables (e.g., growth velocity, and cognitive indices) were assessed using the Spearman Rank-Order correlation. To identify predictors of cognitive outcomes, multiple linear regression models were fitted including birth parameters (birth weight, length, and head circumference) as independent variables. For the subgroup of rhGH treated SGA, additional regression models were computed to evaluate the potential influence of rhGH dose and treatment duration on IQ and its subscales.

The magnitude of the observed associations was quantified using Cohen’s f as the common effect size metric. For the multiple regression analysis, Cohen’s f was obtained from R^2^ using the formula f = √(R^2^/(1 − R^2^)); for the non-parametric comparisons conducted via the Kruskal–Wallis test, the H-statistic was first converted into an eta-squared (η^2^_H_) estimate and then transformed into Cohen’s f to maintain a standardized interpretative framework.

To determine the required sample size based on the primary outcome, a power analysis was conducted, assuming a large effect size (f = 0.50) and a statistical power of 80% for a three groups comparison. The results indicated a sample size of 42 patients, which was increased by 15% (Asymptotic Relative Efficiency) to account for the use of a non-parametric test, bringing required sample size to 48 patients (16 per group) to detect such an effect. The level of statistical significance was set at *p* < 0.05 for all tests.

## 3. Results

### 3.1. Characteristics of the Study Population

Forty-one children born at term were included in the study: 18 SGA children (9 males) and 23 AGA children (15 males). Within SGA group, 10 children (6 males) were treated with rhGH with an average age at treatment of 9.45 ± 2.31 and a mean treatment duration of 2.42 ± 1.85 years.

The general characteristics of enrolled subjects are summarized in Table 1.

### 3.2. Cognitive Assessment (WISC-IV)

Mean index scores for each group are reported in Table 2.

Kruskal–Wallis One Way Analysis of Variance on Ranks revealed a statistically significant difference in IQ among SGA and AGA children (*p* = 0.011), with post hoc comparisons indicating lower IQ scores in the rhGH treated SGA children compared with the AGA group. Furthermore, the analysis of the four cognitive indices revealed a significant difference specifically in the PRI and PSI scores between the SGA and AGA groups.

No statistically significant differences in VCI or WMI scores were found between AGA and SGA children.

**Figure 1:**
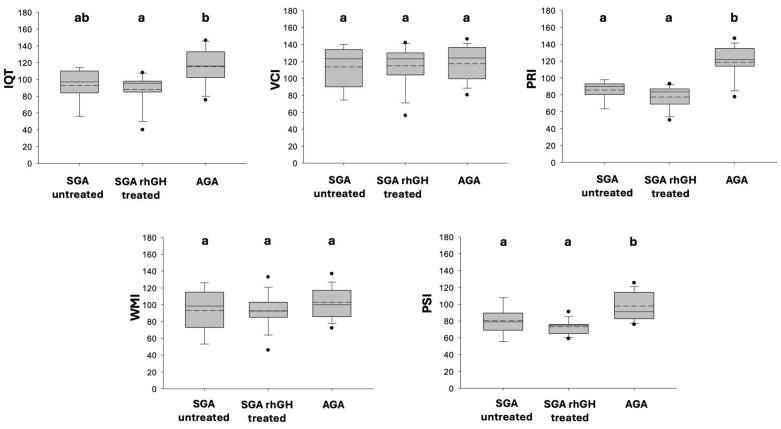
Boxplots showing a visual comparison of the cognitive profiles, including of IQT (total intelligence quotient), VCI, PRI, WMI and PSI, evaluated for all patients during the cognitive assessment. The mean and median values are marked by a dotted line and continuous line, respectively. Statistical significance (*p* < 0.05) is indicated by different superscript letters above the boxes: groups that do not share a common letter are significantly different from one another, whereas groups sharing at least one common letter are not significantly different. Specifically: IQT: the AGA group (b) is significantly different from the rhGH-treated SGA group (a), while the untreated SGA group (ab) shows no significant difference from either. PRI and PSI: the AGA group (b) is significantly different from both SGA subgroups (a). VCI and WMI: no significant differences were found among the groups (all labeled with ‘a’).

A visual comparison of the cognitive profiles of the sample is shown in Figure 1.

### 3.3. Behavioral Functioning

The Mann–Whitney U test comparing of the CBCL composite clinical T-scores between SGA e AGA subgroups showed a statistically significant difference in internalizing symptoms, with higher scores found in the SGA group, median 68 (58 ÷ 78) compared to the AGA group median 50 (50 ÷ 63.5); (*p* < 0.001)). Conversely, no statistically significant difference was observed for externalizing symptoms, with similar T scores in the SGA median 51 (50 ÷ 59) and AGA groups median 50 (50 ÷ 50) (*p* = 0.159), suggesting fewer externalizing symptoms in both cohorts.

### 3.4. Neonatal Predictors of Cognitive Outcome

Multiple linear regression analyses were conducted to explore whether neonatal parameters (birth weight, length, and head circumference) may predict later cognitive outcomes. IQ in our cohort has not been predicted by any linear combination of the parameters considered (R^2^ = 0.280, *p* = 0.190). However, head circumference at birth emerged as a significant predictor of PRI (R^2^ = 0.724, *p* < 0.01), and moderately of PSI (*R*^2^ = 0.327, *p* = 0.045). These last two regression models explained a substantial proportion of the variance. To assess the clinical impact of this effect, Cohen’s f was calculated starting from R^2^ values, yielding a value of 1.62 and 0.69, respectively. Both values indicated a large effect as they overcome the classical threshold of 0.40. So, beyond their statistical significance, these associations suggested meaningful clinical importance, as reflected by their large effect sizes.

### 3.5. Effect of rhGH Treatment

Two separate linear regression models were fitted to further examine whether cognitive outcomes in rhGH treated SGA children were associated with GH dose or treatment duration. Neither model indicated a significant relationship between treatment and IQ (R^2^ = 0.193, *p* = 0.204 for rhGH dose, R^2^ = 0.00975, *p* = 0.786 for treatment duration).

Given the small sample size of rhGH treated group, low statistical power, and non-normal residuals, these findings should be interpreted cautiously, as potential true effects might remain undetected due to the limited dataset.

## 4. Discussion

Being born SGA has been associated with alterations in neurocognitive development and with behavioral disorders, although findings in the literature remain heterogeneous and sometimes conflicting. In our cohort, children born SGA exhibited lower cognitive performance than their AGA peers, as evidenced by significantly lower IQ scores. This finding is consistent with previous studies reporting that children born SGA show poorer cognitive outcomes and an increased risk of intellectual impairment during childhood [7,8], with these differences persisting into adulthood [25].

Among specific cognitive domains, we found a significant difference in PRI scores, with the SGA group performing worse. The PRI measures non-verbal reasoning, visuospatial processing, and the capacity to identify patterns and apply fluid reasoning to novel tasks.

These functions depend primarily on the integrity of the parietal cortex. Neuroimaging studies have demonstrated increased cortical thickness in SGA children [6]. Importantly, this greater cortical thickness does not indicate more advanced development; rather, it is thought to reflect an altered pattern of cortical maturation, characterized by reduced synaptic pruning or atypical cortical layering [26]. Similarly, PSI, which reflects rapid and efficient visual information processing and visuomotor coordination, was significantly lower in our SGA group. This finding is consistent with the results of Tanis et al. [13], who reported lower visuomotor integration scores in SGA children compared with their AGA peers, indicating more pronounced impairment in visuomotor abilities. These abilities rely not only on the posterior parietal cortex but also on major white-matter pathways, the integrity of which may be compromised in SGA children. Neuroimaging studies have demonstrated a substantial reduction in white-matter volume in this population, accompanied by alterations in both cortical and subcortical gray-matter structures [26]. The absence of significant differences in VCI and WMI among SGA and AGA children suggests that verbal and working memory abilities are relatively more preserved compared to visuospatial skills. Nonetheless, even when overall cognitive performance remains within the normal range, systematic reviews identify SGA status still represents a subclinical risk factor for academic and language-related difficulties, including lower scores in vocabulary and reading tasks [16].

The rhGH-treated group achieved lower numerical scores in IQ, PRI, and PSI compared to untreated SGA peers. This observation likely linked to the fact that children who undergo rhGH therapy are typically those who fail to achieve spontaneous catch-up growth. Consequently, this subgroup may represent individuals with a more severe phenotype of growth restriction, which is associated with more pronounced neurocognitive risks. However, the small number of rhGH treated children in our study population limits the strength of these findings.

Notably, SGA individuals are reported to have up to a fourfold higher risk of scoring within the abnormal range on attentional control tasks compared with AGA peers [18,27]. Such attentional difficulties, often accompanied by learning problems, may persist into adolescence and likely reflect functional impairments in fronto-striatal networks, which are essential for efficient attention regulation and executive control.

Data from the CBCL in our cohort showed a statistically significant increase in internalizing symptoms among SGA patients.

Evidence from longitudinal cohorts and meta-analyses indicates that SGA status confers a modest yet reliable elevation in risk for internalizing problems across development [11,28]. This risk is thought to stem from subtle neurodevelopmental alterations associated with fetal growth restriction, including slower information processing, reduced efficiency in emotion regulation, and changes in fronto-limbic maturation and HPA axis stress responsivity [11,28,29]. Such a profile—characterized by greater cognitive inhibition relative to impulsivity—may translate into fewer externalizing behaviors but heightened susceptibility to internalizing symptoms [27,30], particularly under conditions of academic or social stress. Contextual factors may further shape developmental trajectories. In our cohort receiving structured clinical follow up and benefiting from relatively high parental educational and socioeconomic backgrounds, early detection and management of emerging difficulties may help mitigate behavioral dysregulation. Nevertheless, given the potential for later-emerging challenges in behavioral regulation, self-control, or attention—particularly during adolescence—ongoing longitudinal monitoring remains advisable. In our cohort, head circumference at birth emerged as the only neonatal parameter predicting PRI and PSI scores, highlighting the influence of fetal brain growth. This finding aligns with observations in other medical conditions characterized by microcephaly, as well as with longitudinal evidence showing that impaired head growth—reflecting altered fetal brain development—serves as a strong predictor of adverse cognitive outcomes [31]. Among our cohort of SGA children, we found no significant association between rhGH treatment—whether considering dosage or duration—and IQ outcomes. The absence of significant correlations between cognitive outcomes and rhGH treatment, dosage or duration, supports the hypothesis that these differences stem from baseline clinical severity rather than the treatment itself.

## 5. Conclusions

Our findings indicate that the cognitive profile of SGA children born at term is characterized by a slightly reduced overall IQ and selective weaknesses in visuospatial, attentional, and processing speed domains, while verbal and memory skills remain relatively preserved. Early identification of deficits in perceptual reasoning and processing speed may help guide timely cognitive and educational interventions, potentially improving long-term academic and social outcomes.

The principal strengths of this study include the homogeneity of the sample (children born at term, with preterm and syndromic cases excluded), the use of standardized cognitive assessment through the WISC-IV, and the integration of anthropometric, biochemical and behavioral data.

The main limitation of this study is the small sample size. The final cohort included 41 patients, approaching the estimated sample size requirement. However, recruitment of SGA patients was constrained by their clinical characteristics and the application of strict inclusion criteria. This resulted in a slight group imbalance, which may have marginally reduced the statistical power of post hoc pairwise comparisons, although the overall model remained sufficiently sensitive to detect significant global differences.

Additionally, the AGA control group was recruited from a Child Neuropsychiatric Clinic and, although no neurodevelopmental disorders were diagnosed, their referral context may differ from that of a community-based population, potentially introducing selection bias. Nevertheless, this recruitment strategy does not undermine the validity of the observed findings but rather warrants caution in their generalization.

Longitudinal studies with larger samples will be essential to elucidate the mechanistic pathways linking early growth restriction, brain development, and cognitive performance and to determine whether rhGH therapy exerts domain-specific neurocognitive effects or interacts with early catch-up growth trajectories.

## Figures and Tables

**Table 1:** General characteristics of study population. SGA: Small for Gestational Age, AGA: Appropriate for Gestational Age, SDS: Standard Deviation Score, BMI: Body Mass Index.

	SGA Subjects (*n*° = 18)	AGA Subjects (*n*° = 23)	*p* Value
Gender (M/F)	9/9	15/8	-
Gestational age (weeks)	37.9 ± 1.4	39.6 ± 1.3	=0.174
Birth weight (kg)	2.2 ± 0.4	3.2 ± 0.5	<0.001
Birth weight SDS	−2.26 ± 0.63	−0.06 ± 0.80	<0.001
Birth length (cm)	45.1 ± 2.5	50.0 ± 1.6	<0.001
Birth length SDS	−2.07 ± 0.66	0.08 ± 0.85	<0.001
Head circumference (cm)	32.1 ± 1.8	34.8 ± 1.3	<0.001
Head circumference SDS	−1.50 ± 1.06	0.34 ± 1.11	<0.001
At recruitment			
Age (yrs)	10.8 ± 2.8	11.5 ± 4.2	=0.578
Weight (kg)	29.7 ± 9.1	50.9 ± 20.6	<0.001
Weight SDS	−1.81 ± 1.42	−0.06 ± 1.23	=0.003
Height (cm)	130.4 ± 14.2	152.2 ± 17.0	<0.001
Height SDS	−2.05 ± 0.88	0.56 ± 1.15	<0.001
BMI (kg/m^2^)	17.1 ± 3.2	21.1 ± 5.6	=0.011
BMI-SDS	−0.91 ± 1.32	0.46 ± 1.39	=0.032

**Table 2:** WISC-IV mean results. Comparison of cognitive indices across groups was performed using the Kruskal–Wallis one-way analysis of variance on ranks. Statistical significance (*p* < 0.05) is indicated by different superscript letters above the boxes: groups that do not share a common letter are significantly different from one another, whereas groups sharing at least one common letter are not significantly different. SGA: Small for Gestational Age, AGA: Appropriate for Gestational Age, IQT = total Intelligence Quotient; VCI = Verbal Comprehension Index; PRI = Perceptual Reasoning Index; WMI = Working Memory Index; PSI = Processing Speed Index.

	Untreated SGA Children (*n*° = 8)	rhGH Treated SGA Children (*n*° = 10)	AGA Children (*n*° = 23)	*p* Value
IQT	92.75 ± 23.22 ^ab^	88.00 ± 21.57 ^a^	115.30 ± 23.20 ^b^	*p* = 0.011
VCI	113.50 ± 26.38 ^a^	114.80 ± 26.60 ^a^	117.16 ± 21.16 ^a^	*p* = 0.972
PRI	85.62 ± 13.15 ^a^	77.40 ± 14.40 ^a^	118.79 ± 20.98 ^b^	*p* < 0.001
WMI	93.25 ± 27.99 ^a^	92.80 ± 22.37 ^a^	102.94 ± 20.57 ^a^	*p* = 0.422
PSI	80.37 ± 18.82 ^a^	73.20 ± 9.24 ^a^	97.84 ± 17.30 ^b^	*p* < 0.001

## Data Availability

The original contributions presented in this study are included in the article. Further inquiries can be directed to the corresponding author.
